# Dynamic Fusion of Nucleic Acid Functionalized Nano-/Micro-Cell-Like Containments: From Basic Concepts to Applications

**DOI:** 10.1021/acsnano.3c04415

**Published:** 2023-08-07

**Authors:** Zhenzhen Li, Jianbang Wang, Michael P. O’Hagan, Fujian Huang, Fan Xia, Itamar Willner

**Affiliations:** † The Institute of Chemistry, The Center for Nanoscience and Nanotechnology, The Hebrew University of Jerusalem, Jerusalem 91904, Israel; ‡ State Key Laboratory of Biogeology and Environmental Geology, Engineering Research Center of Nano-Geomaterials of Ministry of Education, Faculty of Materials Science and Chemistry, China University of Geosciences, Wuhan 430074, People’s Republic of China

**Keywords:** liposome, biophysics, protocell, sensor, nitrobenzyl phosphate, patterning, biocatalysis, photodeprotection

## Abstract

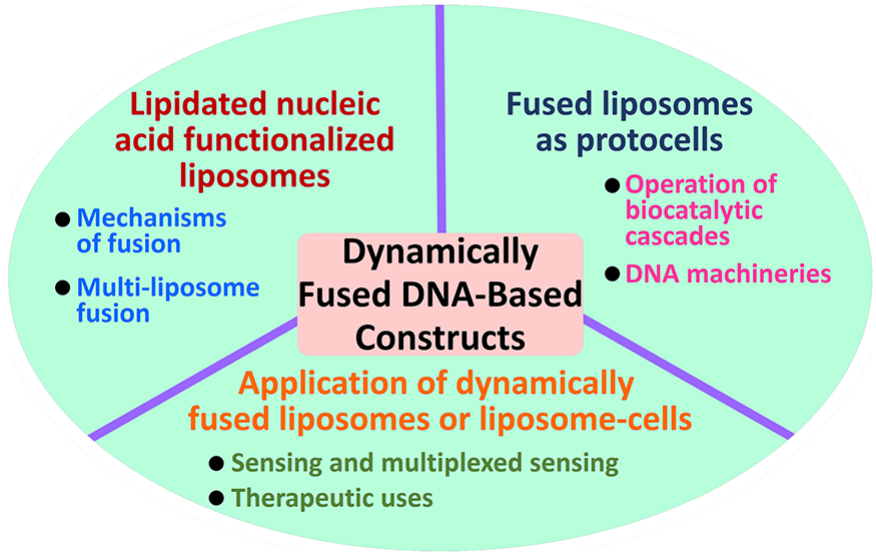

Membrane fusion processes play key roles in biological transformations, such as endocytosis/exocytosis, signal transduction, neurotransmission, or viral infections, and substantial research efforts have been directed to emulate these functions by artificial means. The recognition and dynamic reconfiguration properties of nucleic acids provide a versatile means to induce membrane fusion. Here we address recent advances in the functionalization of liposomes or membranes with structurally engineered lipidated nucleic acids guiding the fusion of cell-like containments, and the biophysical and chemical parameters controlling the fusion of the liposomes will be discussed. Intermembrane bridging by duplex or triplex nucleic acids and light-induced activation of membrane-associated nucleic acid constituents provide the means for spatiotemporal fusion of liposomes or nucleic acid modified liposome fusion with native cell membranes. The membrane fusion processes lead to exchange of loads in the fused containments and are a means to integrate functional assemblies. This is exemplified with the operation of biocatalytic cascades and dynamic DNA polymerization/nicking or transcription machineries in fused protocell systems. Membrane fusion processes of protocell assemblies are found to have important drug-delivery, therapeutic, sensing, and biocatalytic applications. The future challenges and perspectives of DNA-guided fused containments and membranes are addressed.

## Introduction

Cell-cell membrane fusion plays key roles in diverse biological transformations, such as neurotransmission,^1,2^ exocytosis and endocytosis,^3^ signal transduction,^4−6^ cell divisions,^7,8^ and viral infections.^9^ Cell fusion includes two steps where the first step involves the interaction of the membranes in spatial proximity that overcomes steric and/or electrostatic perturbing forces.^3^ Subsequently, the spatially interacting membrane boundaries are exchanged to form intermediate higher curved structures that merge into a fused containment loaded with the mixture of loads present in the fused reservoirs.^10^ In nature, membrane fusion is regulated by a number of proteins or protein subunits, e.g. N-ethylmaleimide-sensitive-factor attachment receptors, SNAREs.^11−14^ Native fusion leads to the delivery of cellular payloads and reactive agents. For example, neuronal fusion in which Ca^2+^-triggered release of neurotransmitters at synapses through fused vesicles is stimulated by SNAREs and proceeds on a submillisecond time scale.^15^ Not surprisingly, beyond increased efforts to understand the biological processes and the principles underlying the supramolecular recognition interaction in native membranes, substantial interests have been directed to the development of synthetic model systems and materials mimicking biological fusion events. One approach involves the synthesis of liposomes in which bioactive fusogenic proteins are integrated within artificial lipid vesicles as functional containments guiding fusion processes.^16−22^ An alternative approach involves the docking of liposomes or membrane-like assemblies with molecular constituents exhibiting complementary supramolecular recognition functionalities, allowing interliposome or intermembrane fusion to proceed. For example, the integration of coiled-coil forming peptides into liposomes led to fusion of the liposomes^23−25^ and docking of liposomes with cholesterol-terminated or lipidated complementary nucleic acid functionalized liposomes led to the fusion of these containments.^26−29^ The membrane properties, such as thickness, rigidity and size of the containments, control the efficacies of fusion.^30,31^

The application of nucleic acid functionalized membranes or liposomes is particularly attractive since the DNA biopolymers provide a means to trigger, and dynamically activate the fusion of the membranes. The information encoded in the base sequence allows the dynamic reconfiguration of the DNA structures. The control over the stability of duplex nucleic acids guided by the number and nature of base pairs^32,33^ and the dynamic strand displacement processes of double-stranded DNA^34^ are key recognition motifs to induce membrane fusion. In addition, the reversible reconfiguration of single-stranded nucleic acids into quadruplexes,^35−37^ the metal-ion-assisted stabilization of nucleic acid based complexes,^38,39^ the assembly of triplexes,^40^ and the stabilization of duplex nucleic acids by intercalated *trans*-azobenzene photoisomerizable units^41−43^ provide structural motifs to trigger the connection of membrane interfaces and induce dynamic fusion. Also, the caging of nucleic acid structures, e.g. hairpins, by photoresponsive *o*-nitrobenzylphosphate ester groups introduced means for the spatiotemporal uncaging of nucleic acid structures integrated in liposome containments, allowing their light-triggered fusion with complementary nucleic acid constituents associated with neighboring liposomes or membrane interfaces.^44^

Besides modeling biological fusion processes, important practical applications may emerge from the fusion machinery. These include the use of the fusion mechanism as a platform for the development of sensors,^45−47^ the delivery and spatiotemporal release of drugs or genes into cells for therapeutic applications,^48−51^ and particularly the development of artificial cells, “protocells”.^52−54^ Diverse protocell containments have been reported in the past decade, including liposomes,^55,56^ polymersomes,^57,58^ dendrosomes,^59^ proteinsomes,^60,61^ hydrogel microcapsules,^62,63^ and aqueous droplets.^64^ Different catalytic,^65,66^ photocatalytic,^67^ and biocatalytic^68^ transformations were driven within these cell-like containments.

The present review addresses recent advances in the development of dynamically triggered fusion of nucleic acid functionalized liposomes to yield integrated cell-like containments and the dynamic fusion of nucleic acid modified liposomes with cell membranes to yield composite functional membranes. Diverse applications of the systems are introduced, and future perspectives of the fields are addressed.

## Fusion of Nucleic Acid Modified Liposomes

**Figure 1:**
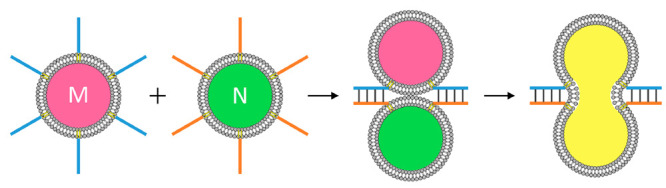
Schematic fusion of two nucleic acid functionalized phospholipid vesicles M and N, where the nucleic acid residues exhibit structural complementarity.

The functionalization of membranes, phospholipid vesicles, or liposomes with nucleic acids functionalized with hydrophobic moieties, e.g., cholesterol-modified nucleic acids, provides a general means to induce the fusion of the liposome carriers.^69^ The recognition properties of nucleic acids, e.g., hybridization of complementary duplex strands^34^ and formation of supramolecular complexes, such as triplex DNA structures, G-quadruplexes, i-motifs^35−37^ or intercalator-stabilized duplexes,^41^ provide a general means to stimulate the fusion of liposomes. By mixing liposomes functionalized with nucleic acids exhibiting complementary recognition features, a liposome complex exhibiting spatial proximity may be formed, leading to the intermixing of the liposome boundary junction and the subsequent mechanical separation of the interlinked boundary into an intact fused liposome where the contents of the liposomes are mixed, as schematically outlined in Figure 1.

This general principle of liposome fusion raises several basic issues: (i) The nucleic acid interaction of the liposomes may lead to geminally bound liposomes with localized loading containments or to fused intact liposomes comprising mixed loading containments. Thus, the mode of interactions in interconnected liposomes and the “real” event of fusion and fusion yield are important topics to consider. (ii) The development of physical tools to follow the dynamic and temporal fusion processes is important. Different methods including dynamic light scattering, microscopy (such as TEM, SEM, or confocal microscopy) optical methods, following the exchange of loaded constituents upon fusion, and chemical or biocatalytic transformations emerging from the exchange of constituents between the liposomes upon fusion were employed to probe the dynamics of liposome fusion and to evaluate the yields of fused liposomes. (iii) The nucleic acid functionalities guiding the fusion processes are usually associated with the membrane boundaries by anchoring the nucleic acids to hydrophobic bridging units, such as cholesterol, tocopherol, or lipids.^70−76^ Moreover, the nature of the nucleic acid bridging units,^28,87,77^ their modes of linkage to the hydrophobic membrane motifs, and their compositional distribution in the interacting membranes play key roles in the fusion efficacies of the synthetic membranes.^30,31,78−80^ (iv) The triggered reconfiguration of the nucleic acid bridges interconnecting the liposomes by stimuli (e.g., light) plays an important role in controlling the fusion efficiency of the liposomes. (v) The practical application of liposome fusion is a major aspect to consider. In the subsequent sections, these different issues will be addressed by examples demonstrating the nucleic acid triggered fusion of liposomes, the application of physical and chemical means to follow the fusion processes, the demonstration of cascaded and triggered fusion of several kinds of liposomes, and discussion of possible applications of fused liposome assemblies.

### Biophysical Insight into Nucleic Acid Based Membrane Fusion

The diverse physical parameters controlling the fusion of synthetic membranes, including the relative sizes of the fusing containments, the composition of the fused boundaries, and particularly the structure (strand lengths) and geometrical patterns of the nucleic acids guiding the fusion process, introduce a blend of biophysical effects that could enhance the efficacy of nucleic acid functionalized synthetic membrane fusion. Indeed, several recent studies contributed basic biophysical insight into the understanding of the fusion processes.

**Figure 2:**
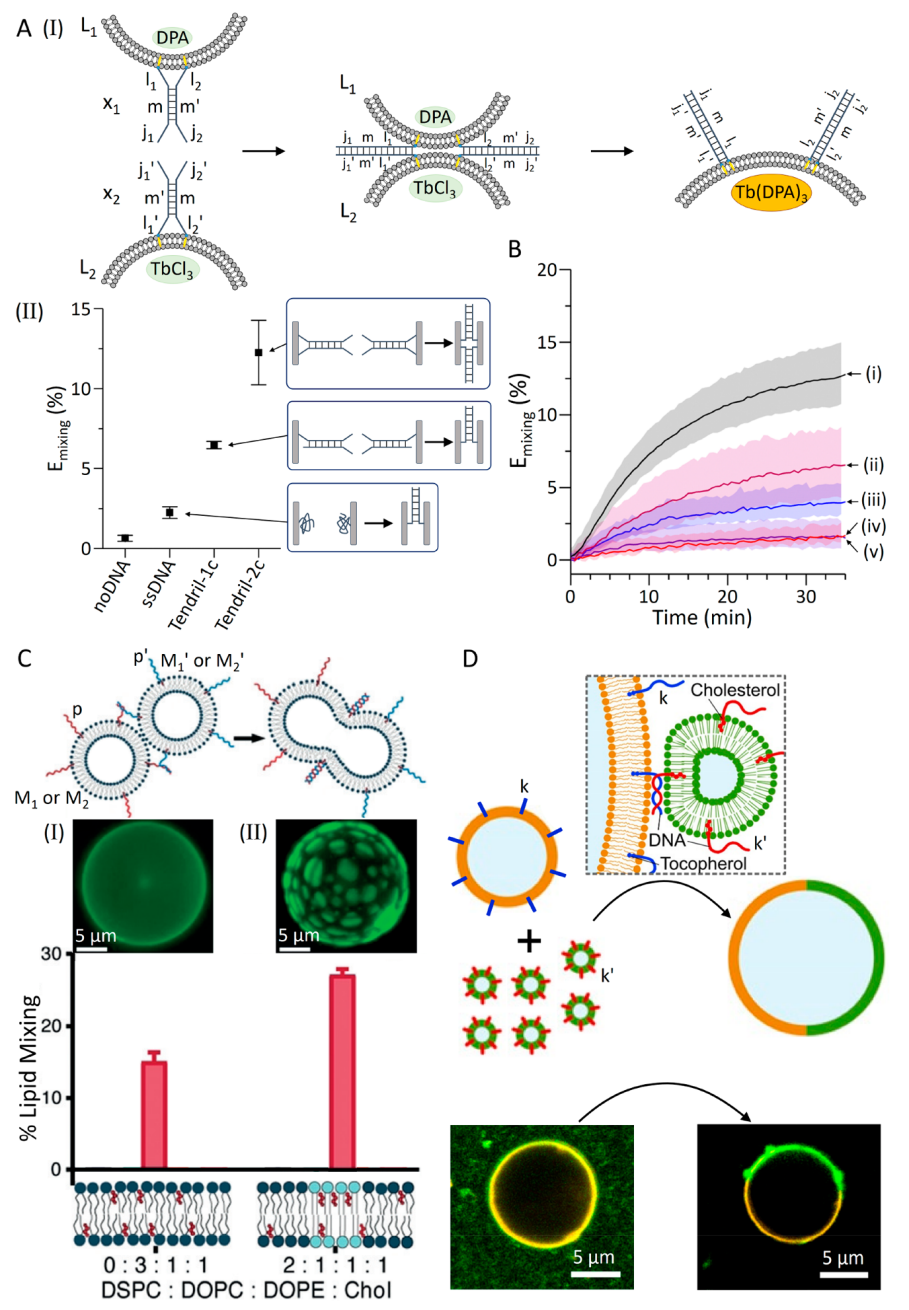
(A) Panel I: schematic fusion of nucleic acid functionalized liposomes, L_1_ and L_2_, modified with tendril DNA structures. Liposome L_1_ is loaded with DPA and liposome L_2_ is loaded with TbCl_3_. The fusion is followed by fluorescence of the Tb(DPA)_3_ complex formed upon mixing the loads in the fused containments. Panel II: fusion efficiencies observed using different nucleic acid DNA tendril configurations upon fusion of the two liposomes. (B) Fusion efficiencies of two nucleic acid functionalized liposomes modified with tendril DNA structures consisting of different lipidated compositions of the bilayer liposome boundaries: (i) 50/25/25 DOPC/DOPE/Chol; (ii) 50/25/25 DOPC/POPE/Chol; (iii) 50/25/25 DOPC/OA/Chol; (iv) 50/25/25 DOPC/DOPC/Chol; (v) 50/25/25 DOPC/POPA/Chol. Reprinted with permission under a Creative Commons CC BY 3.0 License from ref (81). Copyright 2022, Royal Society of Chemistry. (C) Effects of bilayer lipid compositions on the nucleic acid stimulated lipid mixing in the fused liposome containment followed by confocal fluorescence microscopy: (Panel I) bilayer compositions DOPC, DOPE, and Chol at a ratio of 3/1/1; (Panel II) phase segregated compositions DSPC, DOPC, DOPE and Chol at a ratio of 2/1/1/1. Reproduced with permission from ref (77). Copyright 2019, Wiley-VCH. (D) Schematic fusion of small-sized nucleic acid functionalized vesicles with giant-sized nucleic acid modified vesicles demonstrating the spatiotemporal dictated patterned fusion of the small-sized vesicles on the interface boundary of the giant vesicles. Reprinted with permission under a Creative Commons CC BY License from ref (82). Copyright 2021, Wiley-VCH GmbH.

The significance of the pattern engineering of the nucleic acid constructs associated with the interacting liposomes is exemplified in Figure 2A,^81^ where two liposomes L_1_ and L_2_ are functionalized with nucleic acid tendril structures consisting of duplex domains x_1_ and x_2_ linked to the boundaries of liposomes L_1_ and L_2_ with single stranded tethers l_1_, l_2_ and l_1_′, l_2_′, respectively, and the x_1_ and x_2_ duplexes are terminated with single strand tethers j_1_, j_2_ and j_1_′, j_2_′, respectively. The duplexes x_1_ and x_2_ consist of counter complementary strands m/m′. The interaction between the liposomes L_1_ and L_2_ leads to a modulated stepwise strand displacement recognition process involving four-way branching followed by unzipping strand displacement and migration, generating two integrated duplex bridges composed of (l_1_+m+j_1_)/(l_1_′+m′+j_1_′) and (l_2_+m′+j_2_)/(l_2_′+m+j_2_′) (Figure 2A, Panel I). The loading of the liposomes L_1_ (with dipicolinic acid, DPA) and of L_2_ (with Tb^3+^), leads upon the double-zipped duplex formation to the fused liposomes and exchange of constituents, resulting in the formation and fluorescence of the Tb^3+^-DPA complex as a fusion indicator. The advantages of the double-zipped four-way junction nucleic acid modulated strand displacement process on the fusion yield of the liposomes over control systems including analogous single strand duplexes or a tendril structure linked to liposome boundaries by a single anchoring tether (yielding a single duplex l_1_+m+j_1_/l_1_′+m′+j_1_′) bridge are depicted in Panel II. Evidently, the two anchor tendril architectures reveal superior fusion efficiencies, demonstrating the significance of nanostructure tendril engineering on the communication and the modulation of liposome fusion. Moreover, the DNA-guided fusion efficiencies were controlled by the fusogenic lipid constituents comprising the liposome boundaries. For example, the fusion process described in Figure 2A included liposome boundaries composed of the fusogenic lipids 1,2-dioleoyl-*sn*-glycero-3-phosphocholine (DOPC), 1,2-dioleoyl-*sn*-glycero-3-phosphoethanolamine (DOPE), and cholesterol (Chol) at a ratio corresponding to DOPC/DOPE/Chol 50%/25%/25%. By systematically altering the relative composition of the lipid constituents or by exchanging one of the lipid constituents, the tendril DNA-guided fusion yields of the liposomes were significantly controlled by the lipid composition and lipid nature comprising the liposome boundaries (Figure 2B). For example, high fusion yields were demonstrated with liposomes composed of 50/25/25 DOPC/DOPE/Chol (curve i), and the fusion yields decrease in the presence of liposome boundaries composed of 50/25/25 DOPC/POPE/Chol (curve ii), 50/25/25 DOPC/OA/Chol (curve iii), 50/25/25 DOPC/DOPC/Chol (curve iv), and 50/25/25 DOPC/POPA/Chol (curve v). It was concluded that increasing the proportion of the Chol constituent in the lipid boundary enhanced the fusion efficiency, an effect that was attributed to increased packing stress of the lipids resulting in improved interboundary interactions and eventually interfacial boundary defects. Furthermore, by altering the boundary constituents, the significance of membrane-curvature stress on the fusion efficiency was demonstrated. Moreover, by controlling the rigidity of the tendril-DNA modified liposomes by employing soft and hard bilayer liposome-membrane components, the mechanical properties of the fused liposomes could be programmed.^81^

Moreover, it was found that the composition of the lipid boundary membrane of the interacting nucleic acid functionalized liposomes affects the fusion efficiency and, eventually, leads to a phase segregated domain in the fused liposome structures (Figure 2C).^77^ For example, while the fusion of two liposomes M_1_ and M_1_′ composed of the DOPC, DOPE, and Chol at a ratio of 3:1:1 and functionalized with the complementary nucleic acid p and p′ led to a fusion efficiency of 15% (Panel I), the fusion of liposomes M_2_ and M_2_′ stabilized by the four-constituent boundary lipid components DSPC, DOPC, DOPE, and Chol at a ratio of 2/1/1/1 led to phase segregated fused assemblies with an efficiency of 27% (Panel II).

The fusion of vesicles is controlled not only by the constructs of the nucleic acids bridging constituents or the composition of the boundaries modulating the fusion process but also by the relative sizes of the fusing membrane containments. For example, it was demonstrated that upon fusion of small-sized unilamellar vesicles (SUVs, ca. 100 nm) with giant unilamellar vesicles (GUVs, ca. 10 μm), spatiotemporally guided, domain-dictated fusion of the SUVs proceeds (Figure 2D).^82^ The GUVs were functionalized with tocopherol-tagged nucleic acid (k) and labeled with a yellow fluorescent dye. The SUVs were modified with a cholesterol-tagged nucleic acid (k′). Subjecting the GUV component to the SUVs labeled with the green fluorescent dye results in the site-dictated fusion of the SUV units, where the primary complementary duplex bridging and fusion of the SUV containment promotes and enhances the subsequent fusion of the SUVs to neighboring domains of the initially fused SUVs, resulting in the dictated spatiotemporal fusion of the SUVs on the GUV boundary while eliminating random fusion of the SUVs on the GUV structure. The spatially dictated fusion of the SUVs on the GUVs was demonstrated by the localized green fluorescent image of the fused SUVs. From the resulting green fluorescence intensity, it was estimated that ca. 5600 SUVs were colocalized on the fused domain of the GUVs. The spatially dictated fusion of the SUVs was attributed to the localized tension of the GUV boundary after the first fusion event. Namely, the primary fusion between the SUVs and GUVs lowers the energy barrier of the lipid boundary for a secondary fusion event, thereby leading to the localized patterned fusion of the SUVs. The localized dictated fusion of SUVs with GUVs might have important consequences on the asymmetric division of the GUVs into smaller vesicle containments.

Realizing that fusion between liposomes and the assembly of liposomes carrying functional loads such as vaccines or therapeutics will play an important role in nanomedicine, the biophysical understanding of liposome fusion processes will attract continuous interest.

### Nucleic Acid Based Triggered Fusion of Synthetic Membranes

**Figure 3:**
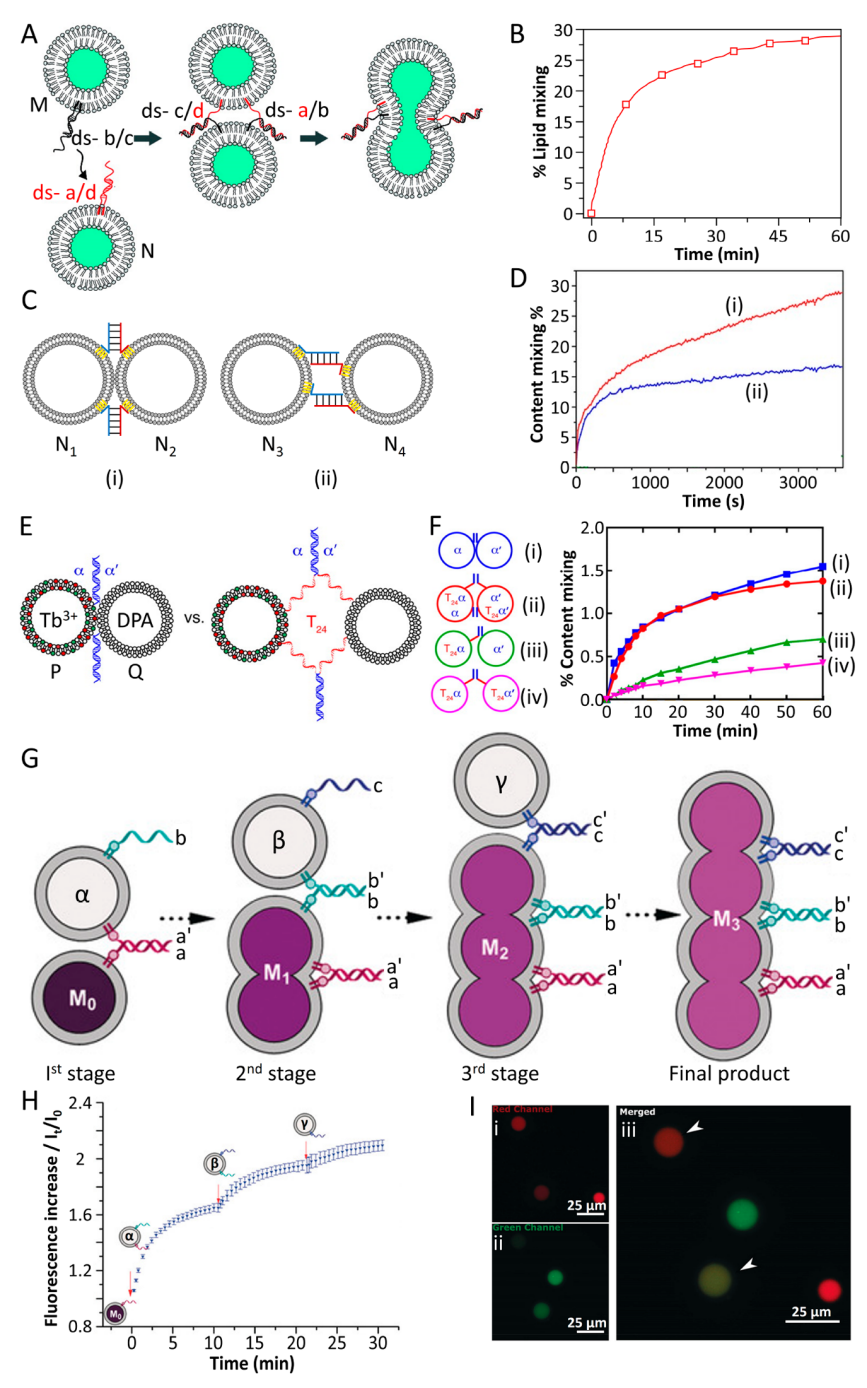
(A) Schematic fusion of two nucleic acid functionalized phospholipid vesicles exhibiting base-pair complementarity. Vesicles are loaded with a donor/acceptor FRET fluorophore pair, and the fusion process is followed by the FRET signal emerging in the fused vesicle. (B) Yield of the fused liposomes evaluated by the emergent FRET signal. Reproduced with permission from ref (28). Copyright 2007, American Chemical Society. (C) Probing fusion efficiency of two nucleic acid functionalized vesicles composed of complementary nucleic acids exhibiting variable base-pair directionalities: (i) zipper orientation mode; (ii) nonzipper orientation mode. (D) Fusion yields corresponding to (i) zipper mode base pair duplex mode and (ii) nonzipper duplex orientation mode. Reproduced with permission from ref (83). Copyright 2017, Wiley-VCH. (E) Effect of bridging tether motifs on the duplex-induced fusion efficiency of two vesicles loaded with Tb^3+^ ions and the DPA ligand. Fusion yields are evaluated by the luminescence intensity of the Tb^3+^-DPA complex in the fused structure. (F) Time-dependent fluorescence changes of the fused vesicles using the bridging fusion motifs (i)–(iv). Reproduced with permission from ref (84). Copyright 2009, Proceedings of the National Academy of Sciences. (G) Stepwise nucleic acid duplex-induced fusion of four different vesicles, where the fusion process is followed by the fluorescence enhancement of the self-quenched SRB probe in vesicle M_0_ upon stepwise fusion-driven dilution of the probe. (H) Temporal fluorescence changes of SRB upon stepwise duplex-induced fusion of M_0_ to M_3_. (I) Confocal fluorescence microscopy images corresponding to the fusion of two giant unilamellar vesicles loaded each with SRB or Atto 647: (i) the Atto 647-loaded vesicle; (ii) the SRB-loaded vesicle; (iii) the merged image of the fused mixture of vesicles. Arrows indicate fused vesicles. Reproduced with permission from ref (85). Copyright 2017, Wiley-VCH.

Figure 3A outlines the schematic fusion of two phospholipid vesicles M and N functionalized with cholesterol-modified nucleic acid duplexes (b)/(c) and (a)/(d). The toehold domains exhibit base complementarity leading to the interconnection and subsequent fusion of the vesicles.^28^ By the integration of a lipidated donor (*Bodipy*500/510-C5-HPC 2-(4,4-difluoro-5-octyl-4-bora-3a,4a-diaza-*s*-indacene-3-pentanoyl)-1-hexadecanoyl-*sn*-glycero-3-phosphocholine) and acceptor (*Bodipy*530/550-C5-HPC 2-(4,4-difluoro-5,7-diphenyl-4-bora-3a,4a-diaza-*s*-indacene-pentanoyl)-1-hexadecanoyl-*sn*-glycero-3-phosphocholine) dyes into the boundary of the vesicles, the resulting FRET signal transduced by the donor/acceptor pair provided a readout signal that probed the fusion process. The fusion resulted in the dilution of the donor and acceptor constituents in the enlarged fused boundary, resulting in the enhanced fluorescence of the donor constituent as a result of lowering the FRET efficiency (Figure 3B). Furthermore, the direction of the duplex-driven fusion of the liposome was found to affect the fusion efficiency^83^ (Figure 3C). Two liposomes N_1_/N_2_ functionalized with lipidated complementary nucleic acids led to a zipper-duplex stimulated fusion, (i), whereas two liposomes N_3_/N_4_ modified with complementary lipidated nucleic acids induced a nonzipper stimulated fusion, (ii). It was demonstrated that zipper-duplex-mediated fusion of the liposomes revealed superior efficiency over the nonzipper-driven fusion (Figure 3D; 29% vs 18% fusion efficiency). In a related study,^84^ the nature of bridging lipidated complementary nucleic acids leading to the interconnection of the vesicles and their subsequent fusion was examined (Figure 3E). Specifically, the effect of spacer nucleic acid units separating the complementary nucleic acid constituents from the liposomes on the interconnection of the vesicles and their subsequent fusion was examined (Figure 3E). The fusion process was followed by loading vesicle P with Tb^3+^ and vesicle Q with DPA and probing the fluorescence of the resulting Tb^3+^/DPA complex generated upon the fusion of vesicles and exchange of loading constituents in the fused liposomes. By applying an appropriate calibration curve, the quantitative evaluation of the dynamic fusion efficiency and content exchange was demonstrated (Figure 3F). While vesicles functionalized with complementary nucleic acid strands lacking spacer units demonstrated effective fusion, the incorporation of spacers perturbed the fusion process.

In addition, cascaded fusion of a series of lipidated nucleic acid functionalized liposomes was accomplished^85^ (Figure 3G). The liposome M_0_ was functionalized with lipidated nucleic acid (a) and treated with liposome α functionalized at the boundary with two nucleic acids (a′) and (b). This resulted in the fusion of M_0_ and α. Subsequently, the fused M_1_ liposome was treated with liposome β modified at the boundary with nucleic acids (b′) and (c), leading to the fused liposome M_2_ that is functionalized with a tether (c). The cascaded treatment of the three-fused liposome M_2_ with liposome γ functionalized with nucleic acid tethers (c′) led to the four liposome cascaded fused containment M_3_. By loading the parent M_0_ liposome with a concentrated, self-quenched sulforhodamine B (SRB) solution, the stepwise cascaded fusion led to the dilution of the dye and the stepwise enhanced fluorescence of the probe dye in the containment, as shown in Figure 3H. Furthermore, the fusion of two giant unilamellar vesicles loaded with SRB or Atto647 dyes was analyzed by confocal laser scanning microscopy (Figure 3I).

Moreover, an important issue that is not fully resolved involves the possible leakage of chemical agents incorporated in the nucleic acid guided fused liposomes. While the integration of nucleic acids modified with a single cholesterol ligand into the liposome bilayer membrane led to fused liposomes revealing substantial leakage of the contents,^27,28^ the functionalization of the fusion-guiding nucleic acids with four lipidated ligands, and the integration of these nucleic acids into the liposome boundaries, led to fused containments without notable leakage of the loads.^83^ This was attributed to the improved anchoring of the nucleic acids to the bilayer boundary by the four lipid tethers that results in more intimate interactions upon the fusion and elimination of possible defects in the fused boundary. While these experiments reveal the effect of structure–function relationships of the interbridging nucleic acid constituents on the “quality” of the fused containments, it is obvious that future systematic studies exploiting the effects of the bilayer membrane environment of the nucleic acid bridging constituents and the functional integrity of the fused membrane and the possible chemical effects of the loads on the leakage are desirable.

**Figure 4:**
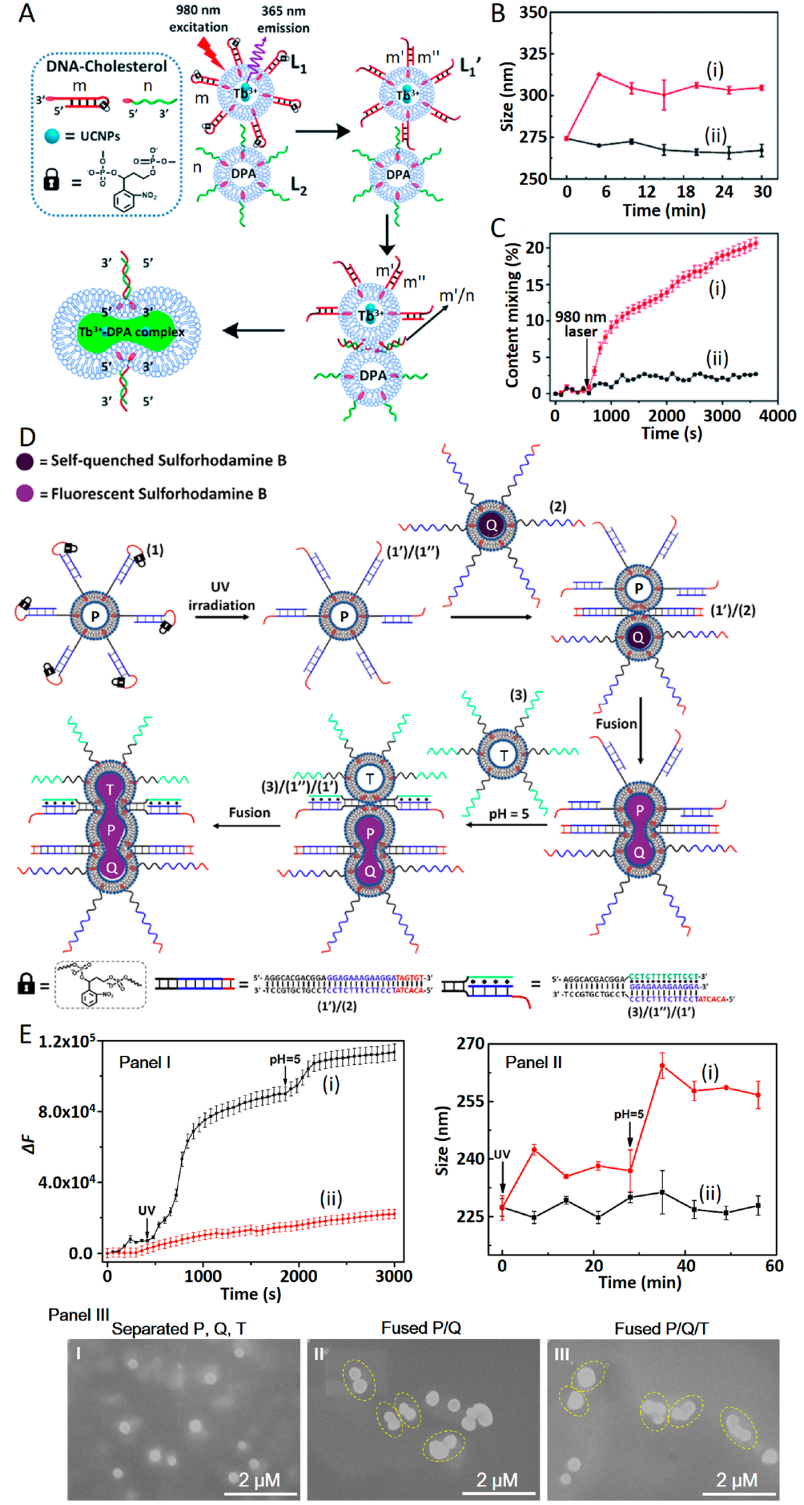
Photochemically triggered fusion of two liposomes through the light-induced uncaging of an *o*-nitrobenzylphosphate ester protected DNA hairpin-functionalized liposome. (B) Following the light-stimulated fusion of the L_1_/L_2_ liposomes by DLS probing of the size-changes: (i) in the presence of light; (ii) without light deprotection of the hairpins. (C) Probing the fusion processes by the temporal fluorescence changes as a result of formation of the Tb^3+^-DPA complex. Reprinted with permission under a Creative Commons CC BY 3.0 License from ref (44). Copyright 2020, Royal Society of Chemistry. (D) Stepwise light-induced and pH-triggered fusion of three nucleic acid functionalized liposomes P/Q/T, where the primary fusion between P and Q involves the light-stimulated uncaging of the *o*-nitrobenzylphosphate DNA hairpin modified liposome P, and the pH-triggered fusion of the fused P/Q assembly with T using pH-stimulated interliposome triplex motif. (E) Following the stepwise three-liposome fusion process by (Panel I) temporal fluorescence changes as a result of dilution of the self-quenched SRB probe, (Panel II) temporal size changes of the fused assemblies evaluated by DLS, and (Panel III) SEM images of the liposomes upon the stepwise fusion. Reprinted with permission under a Creative Commons CC BY License from ref (86). Copyright 2023, Wiley-VCH GmbH.

An alternative method to fuse nucleic acid functionalized liposomes involved the use of light as the fusion trigger^44^ (Figure 4A). A mixture of two liposomes, L_1_ and L_2_, acted as the fusion constituents. Liposomes L_1_ were functionalized with the cholesterol-modified *o*-nitrobenzylphosphate ester photoprotected caged nucleic acid hairpin structure m and loaded with Tb^3+^ and upconversion nanoparticles (UCNPs). Liposomes L_2_ were loaded with DPA, and their boundaries were functionalized with the cholesterol modified nucleic acid n. Near-IR irradiation of the mixture of liposomes resulted in the UCNP-photochemical activation of the UV-responsive *o*-nitrobenzylphosphate ester groups associated with m, resulting in the cleavage of the photoprotected hairpin and the formation of the m′/m″ duplex-modified liposomes L_1_′. The subsequent displacement of the duplex unit m′/m″ associated with L_1_ by the strand n on liposome L_2_ led to the intimate formation of L_1_/L_2_ liposomes bridged by duplex m′/n, resulting in the fusion of the liposomes and the exchange of the loads associated with the two liposomes in the integrated fused containment. The mixing of the constituents led to the formation of a fluorescent Tb^3+^-DPA complex. Accordingly, the light-stimulated fusion of liposomes could be followed by the size changes associated with the fusion process and the dynamic fluorescence changes originating from the formation of the Tb^3+^-DPA complex (Figure 4B,C). The advantage of using light as a fusion trigger, as compared to the duplex-DNA stimulated fusion of liposomes, rests on the fact that a “dormant” fusion-inactive mixture of L_1_/L_2_ can be stored and spatiotemporally activated by light toward fusion.

The light-stimulated triggered fusion process of liposomes has been coupled to a pH-stimulated C-G-C^+^ triplex DNA liposome-bridging motif that allowed the cascaded fusion of three liposomes.^86^ This is exemplified in Figure 4D with the schematic stepwise fusion of the three liposomes P, Q, and T. Liposomes P were functionalized with the cholesterol-modified *o*-nitrobenzylphosphate ester photoactive caged DNA hairpin (**1**), liposomes Q were modified with the cholesterol-functionalized nucleic acid (**2**), and liposomes T were functionalized with a cytosine (C) rich strand (**3**). The UV-light irradiation of the mixture of liposomes P and Q resulted in the photodeprotection and uncapping of the hairpin (**1**) associated with P to yield the (**1′**)/(**1″**) duplex functionalized liposome P. The parent hairpin (**1**) was engineered, however, to yield upon light-stimulated deprotection the duplex (**1′**)/(**1″**) that can be displaced by the strand (**2**) to yield the (**1′**)/(**2**) duplex bridged liposomes P/Q leading to their fusion. The fused liposomes P/Q are, however, functionalized with the duplexes (**1′**)/(**1″**) that under acidic conditions (pH = 5.0) can form a triplex C-G-C^+^ structure between the strand (**3**) and the duplex (**1′**)/(**1″**). The triplex bridge (**1′**)/(**1″**)/(**3**) between the fused P/Q liposome and T leads to the cascaded triple-fused liposome structure P/Q/T. The stepwise fusion of the three liposomes was followed by loading liposomes Q with self-quenched sulforhodamine B dye and monitoring the stepwise fluorescence enhancement upon dilution of the dye as a result of stepwise fusion (Figure 4E, Panel I). The increase of the liposome size was probed by light scattering from the parent size of liposomes P and Q (225 ± 5 nm) to the fused P/Q configuration (240 ± 5 nm) and the enlarged P/Q/T fused structure (265 ± 5 nm) (Panel II), and by SEM imaging of the resulting individual/fused liposomes (Panel III). In fact, the fusion of the three liposomes was accomplished upon the single step triggered fusion of the three separated liposomes by irradiation of the mixture at λ = 365 nm at pH = 5.0. This demonstrated the advantages of the light/pH concomitant fusion process that allowed the spatiotemporal fusion of three liposome containments by the cooperative light/pH triggers.

**Figure 5:**
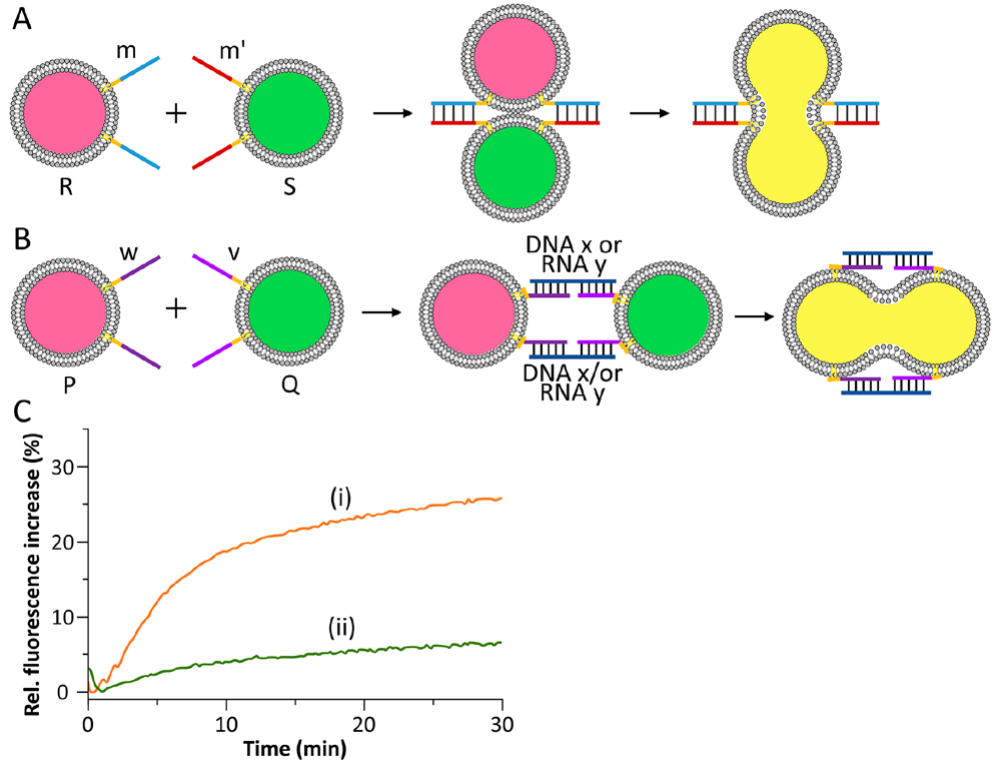
(A) Schematic fusion of two liposomes functionalized with complementary PNA strands. (B) Schematic fusion of liposomes functionalized with noncomplementary PNA strands being bridged by an auxiliary DNA or RNA strand that yield the duplex bridging motif. (C) Fluorescence changes upon (i) interbridging the liposomes by the auxiliary RNA strand and (ii) interbridging the liposomes by the auxiliary DNA strand. Reproduced with permission from ref (87). Copyright 2017, Royal Society of Chemistry.

Besides the bridging and fusion of liposomes by complementary lipidated nucleic acids, synthetic lipidated complementary peptide nucleic acids (PNA) tethered to liposomes were applied to induce the fusion of liposomes.^87^ For example, Figure 5A depicts the schematic zipper-fusion of two liposomes R and S functionalized with lipidated complementary PNA tethers m and m′. By employing two liposomes P and Q functionalized with noncomplementary PNA tethers w and v, the bridging and fusion of the liposomes could be accomplished by an auxiliary tether DNA (x) or RNA (y) (Figure 5B). Interestingly, the fusion yield stimulated by the auxiliary RNA strand (y) corresponded to 25%, whereas the fusion induced by the DNA strand (x) was substantially lower (10%) (Figure 5C). The enhanced RNA-triggered fusion efficiency was attributed to the higher affinity binding interaction with the RNA strands, as compared to the DNA strands, to the PNA tethers associated with the bridged liposomes.

**Figure 6:**
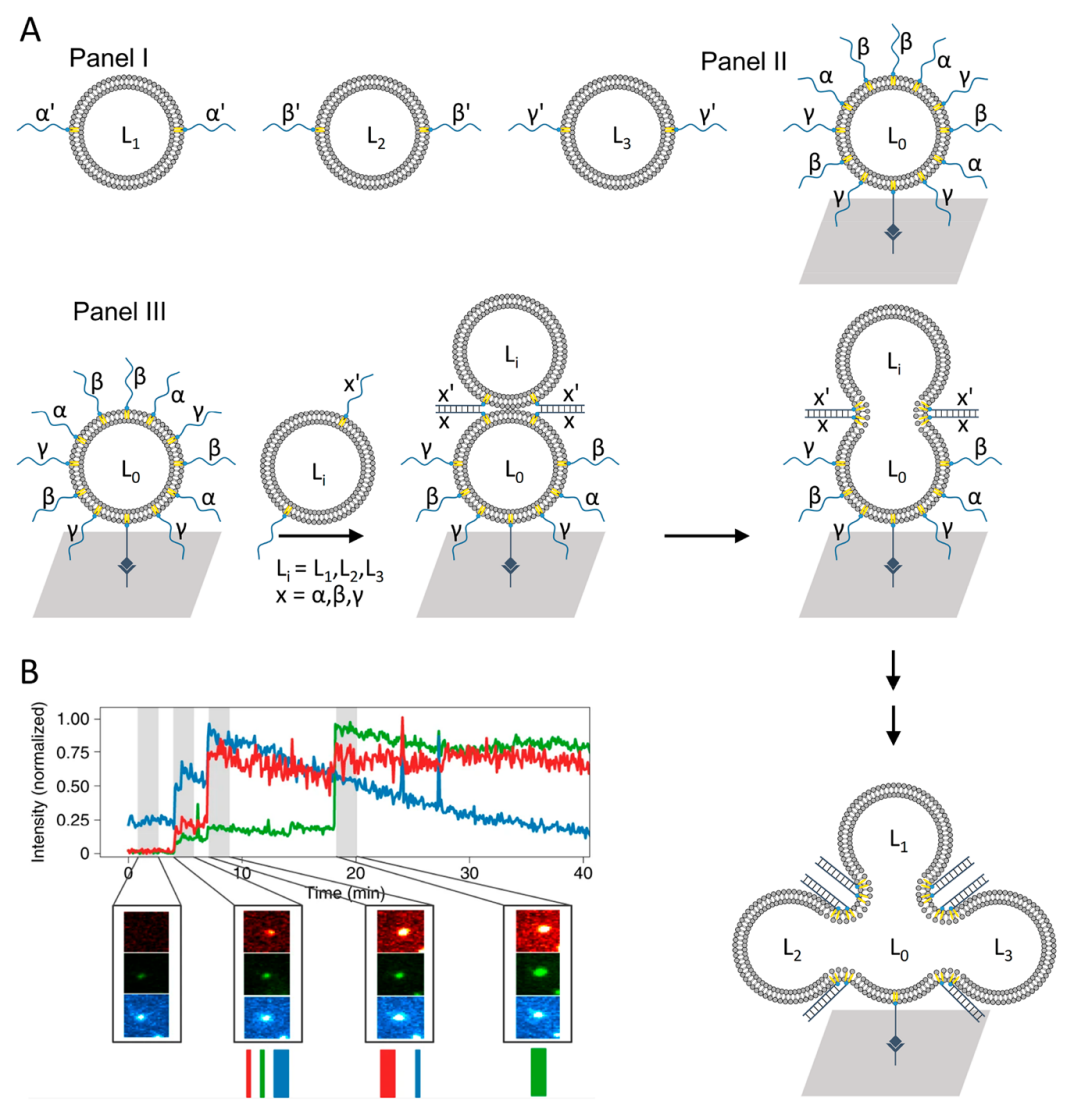
(A) Panel I: three liposomes L_1_, L_2_, and L_3_ modified with nucleic acids α′, β′, and γ′ labeled with variable contents of three different fluorophores. Panel II: schematic assembly of a core liposome L_0_, functionalized with three complementary nucleic acid tethers α, β, and γ, immobilized through a biotin/streptavidin anchor onto a glass slide. Panel III: schematic fusion of the diverse liposomes L_1_, L_2_, and L_3_ with the core liposome L_0_, associated with the surface, using the duplex fusion motifs. (B) TIRF imaging of the fused liposomes using the fluorescence channel associated with the three fluorophores comprising the identifying code of the liposome. Reproduced with permission from ref (88). Copyright 2022, Springer Nature.

An approach to assemble arrays of fused cascaded liposomes exhibiting an imprinted fluorescent barcode identification platform was developed.^88^ This is exemplified in Figure 6A with the dynamic cascaded fusion of three types of liposomes L_1_, L_2_, and L_3_ labeled with three distinct fluorophores, F_1_ (red, ATTO-655-DOPE), F_2_ (green, ATTO-550-DOPE), and F_3_ (blue, 3,3′-dioctadecyloxacarbocyanine perchlorate). Each of these liposomes was modified at its boundary with a different nucleic acid tether α′, β′, and γ′ (Figure 6A, Panel I). A core liposome L_0_ was assembled on a glass slide through boundary-associated biotinylated lipidated nucleic acid that binds to the streptavidin-functionalized glass support. The core liposome construct was functionalized with a dense population of lipidated nucleic acid strands α, β, and γ that allowed the stochastic fusion of the liposomes mixture L_1_, L_2_, and L_3_ to the surface confined liposome L_0_ through the respective duplex bridges α/α′, β/β′, and γ/γ′ (Figure 6A, Panels II and III). The stochastic cascaded fusion of the liposomes L_1_, L_2_ and L_3_ lead, then, to the cascaded multiliposome fused assembly on the core liposome L_0_. Using total internal reflection (TIRF) microscopy, the parallel imaging of the three (red, green, and blue) fluorescence channels and the collection of the temporal fluorescent intensities of the individually developed fused containments were recorded. The temporal evolution of the different fluorescent signals and their intensities provided, then, a characteristic barcode for that fused containment (Figure 6B). That is, the fusion procedure and the TIRF imaging process enable the fabrication of an array of containments, each characterized by a specific barcode pattern. Realizing the resolution of the experimental method, it was suggested that ∼42000 containers per mm^2^ of barcoded fused containments could be fabricated. It should be noted that by the TIRF scanning of the array, the decoding of the specific barcode associated fused containments could be identified. Realizing that the method leads to a mixture of the loads associated with the stochastically fused barcoded containment, the formation of a rich library of defined containments exhibiting compositional diversity can be anticipated. Thus, by examining the collective arrays of fused liposomes and identifying a superior reactivity pattern in a target liposome, decoding of the liposome defines the preferred compositional loading for the reaction target. This procedure is envisaged to be useful for the high-throughput screening of drugs or catalysts.

**Figure 7:**
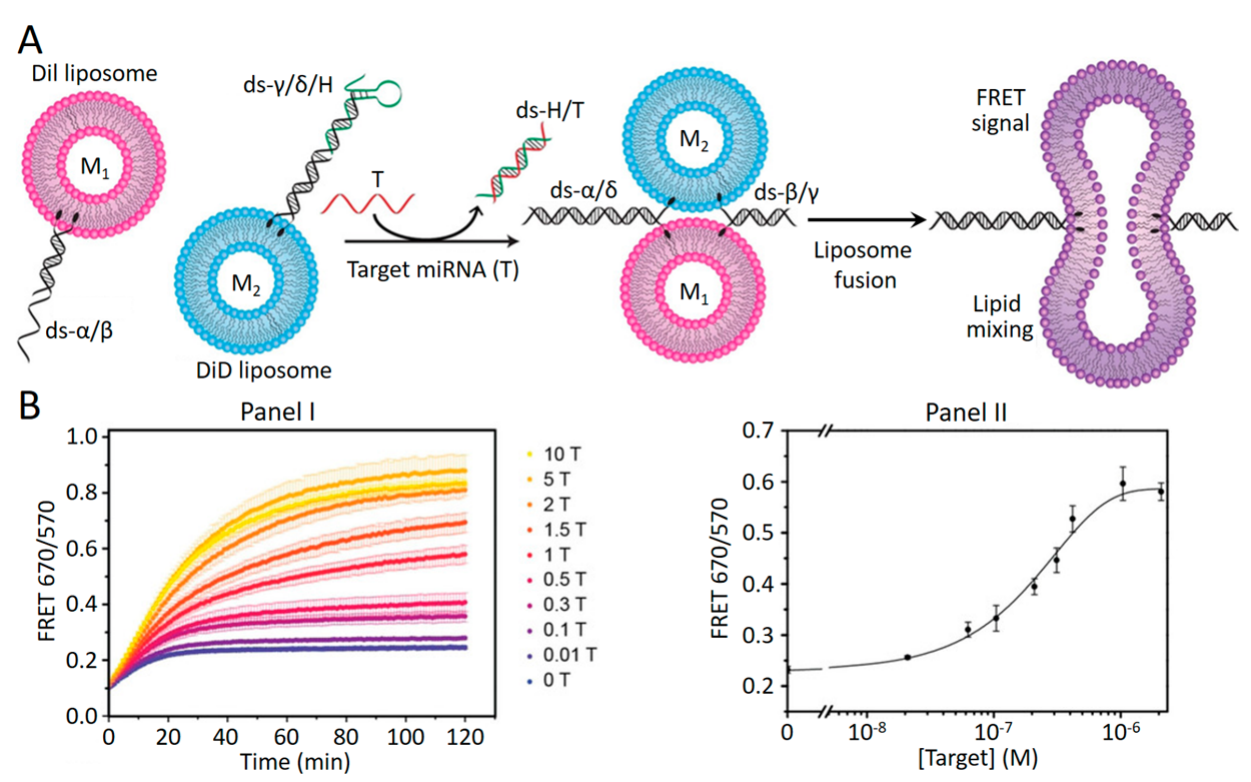
(A) Schematic sensing of miRNA-29a through the miRNA-stimulated fusion of two nucleic acid functionalized liposomes loaded with fluorophore pairs. The FRET pair signal emerging upon the fusion of the liposomes quantitatively transduces the sensing event. (B) Panel I: temporal FRET intensities emerging upon analysis of variable concentrations of the target miRNA-29a. Panel II: derived calibration curve relating the FRET fluorescence intensities to the concentrations of target miRNA-29a. Reprinted with permission under a Creative Commons CC BY License from ref (45). Copyright 2018, Wiley-VCH GmbH.

Different applications of the fusion of liposome assemblies were suggested, including the use of the process for sensing or the organization of cell-like functional containments, “protocells”. Figure 7A outlines the method to apply the fusion of two liposomes for sensing applications,^45^ specifically the detection of microRNA-29a (miR-29a). Two liposomes M_1_ and M_2_ were loaded with the fluorescent dyes 1,1′-dioctadecyl-3,3,3′,3′-tetramethylindocarbocyanine perchlorate (DiIC18(3), DiI) and 1,1′-dioctadecyl-3,3,3′,3′-tetramethylindodicarbocyanine perchlorate (DiIC18(5), DiD). The liposomes M_1_ were modified at their boundaries with cholesterol-modified duplex nucleic acids α/β, where strand α includes a toehold-bearing single-strand tether, and liposomes M_2_ were functionalized at their boundaries with the cholesterol-modified duplexes γ and δ, where nucleic acid δ included a toehold-bearing single strand that was further hybridized with the hairpin nucleic acid structure H that acted as the sensing unit and included in its structure the miR-29a recognition sequence. In the presence of the miR-29a target analyte T, hairpin H is opened and released in the form of duplex T/H. The uncaged strand δ includes in its toehold tether the complementary sequence to the toehold tether of strand α, resulting in the displacement of duplex α/β associated with liposome M_1_ and the formation of α/δ duplex bridged liposomes M_1_ and M_2_. The released strand β is, however, complementary to strand γ associated with liposomes M_2_, leading to the cooperative bifunctional bridging of liposomes M_1_ and M_2_ by duplexes α/δ and β/γ, resulting in the fusion of the liposomes. The mixing of the fluorophore in the membrane of the fused liposomes leads to a FRET fluorescence signal from the DiI fluorophore donor to the DiD fluorophore acceptor. The resulting FRET signal intensity is, however, controlled by the fusion efficiency, and this is dictated by the efficiency of the release of the hairpin H by the analyte miR-29a that is demonstrated by the concentrations of the miR-29a (Figure 7B, Panel I). The derived calibration curve corresponding to FRET signal intensities as a function of the concentrations of miR-29a enabled the selective analysis of the target miR-29a with a detection limit corresponding to 18 nM (Panel II).

**Figure 8:**
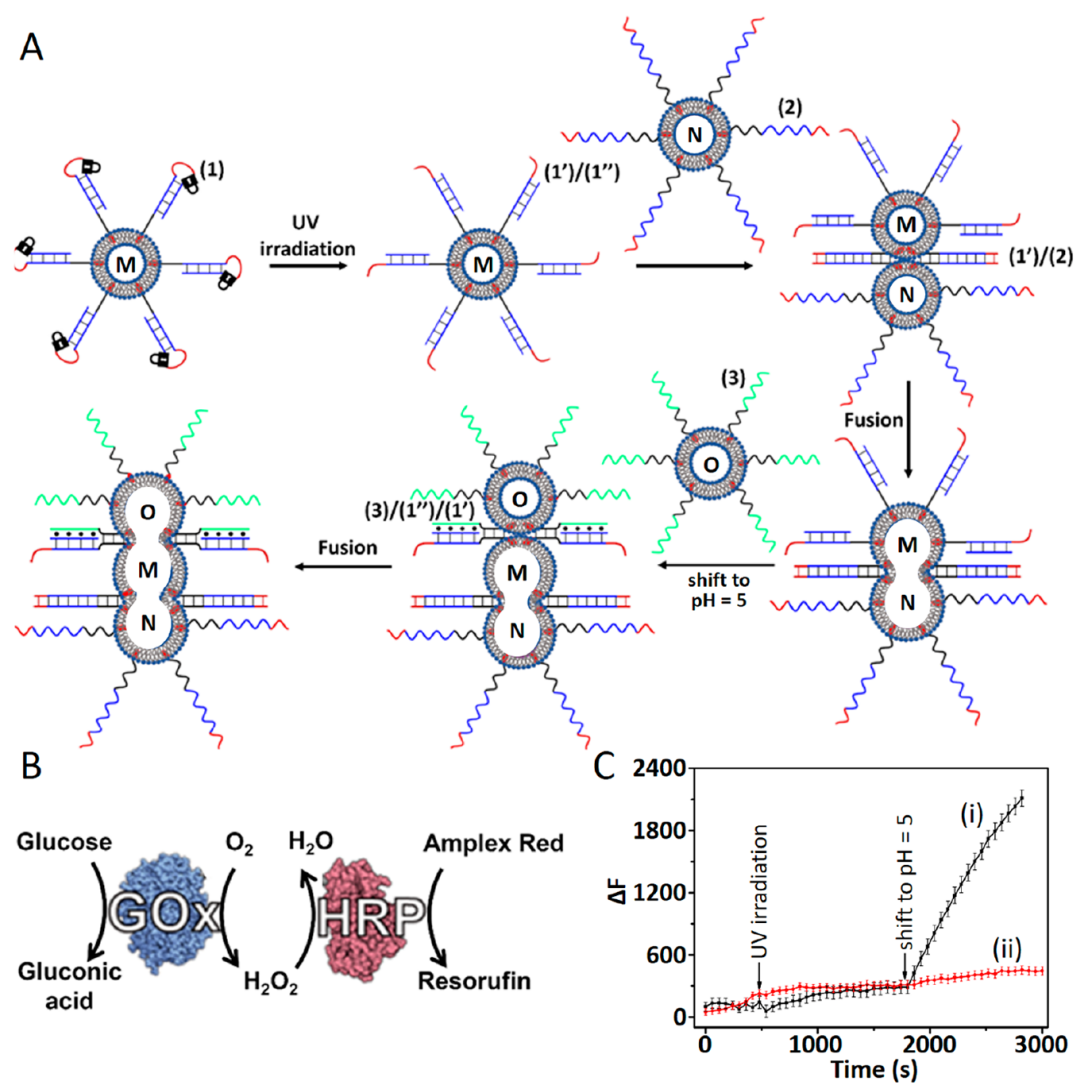
(A) Light- and pH-induced fusion of three liposomes M (loaded with GOx), N (loaded with glucose), and O (loaded with Amplex Red and HRP) using duplex and triplex fusion motifs, for the activation of the GOx/HRP cascade. (B) GOx/HRP biocatalytic cascade involving the GOx-catalyzed aerobic oxidation of glucose to gluconic acid and H_2_O_2_ and the subsequent HRP-catalyzed oxidation of Amplex Red by H_2_O_2_ to yield fluorescent Resorufin. (C) Temporal fluorescence changes of Resorufin upon: (i) the stepwise photoinduced fusion of liposomes M/N and the subsequent pH-stimulated triplex-guided the fusion of liposome O to the fused M+N assembly and (ii) control system in the absence of light-activated deprotection of the hairpin-functionalized liposome M. Reprinted with permission under a Creative Commons CC BY License from ref (86). Copyright 2023, Wiley-VCH GmbH.

The fusion of liposomes and the exchange of biocatalytic loads in the fused containment enabled the assembly of functional cell-like containments in which biocatalytic cascades and biocatalytic machineries operate in analogy to biocatalytic transformations in living cells.^28,50,89−91^ In fact, substantial research efforts have been directed to the development of cell-like micro-/nanocontainments, protocells.^52−54^ Different protocell assemblies were suggested, including liposomes,^55,56^ polymersomes,^57,58^ dendrosomes,^59^ proteinsomes,^60,61^ aqueous microdroplets,^64^ and hydrogel microcapsules.^62,63^ Diverse chemical and biocatalytic constituents were loaded in such artificial cell containments, and catalytic, photocatalytic, and biocatalytic processes were driven in these cell-like containments.^92−95^ The advantages of operating catalytic and biocatalytic cascades in confined cell-like environments, over cascaded transformations in homogeneous phases, were discussed.^96−98^Figure 8A depicts the three-liposome fusion guided activation of the glucose oxidase (GOx)/horseradish peroxidase (HRP) two-enzyme cascade.^86^ The fusion of three liposomes using light and pH as triggers followed an earlier pathway, outlined in Figure 4D. Liposomes M were loaded with the enzyme GOx and functionalized with the cholesterol-modified *o*-nitrobenzylphosphate ester caged hairpin structure (**1**). Liposomes N were loaded with glucose and modified at their boundaries with the cholesterol-modified nucleic acid (**2**), and liposomes O were loaded with HRP and Amplex Red and modified at their boundaries with the cholesterol-functionalized nucleic acid (**3**). Irradiation of the liposome mixture M and N led to the photodeprotection of the *o*-nitrobenzylphosphate ester caged hairpin structure (**1**) and the (**2**)-functionalized liposome-stimulated displacement of the photochemically uncaged duplex (**1′**)/(**1″**) associated with M, leading to the fusion of (**1′**)/(**2**)-bridged liposomes (Figure 8A; see also Figure 4D). The subsequent pH-induced (pH = 5.0) formation of the C-G-C^+^ triplex structures between the fused liposomes M/N functionalized with the (**1′**)/(**1″**) duplex and the (**3**)-modified liposomes O yielded the triplex (**3**)/(**1′**)/(**1″**) structure between fused M-N and O that resulted in the fusion of the three liposomes M/N/O (Figure 8A and Figure 4D) and the mixing of the loads associated with the individual liposomes. Mixing of the contents of the three liposomes led to the activation of the GOx/HRP two-enzyme cascade (Figure 8B), where aerobic GOx-catalyzed oxidation of glucose yielded gluconic acid and H_2_O_2_, and the resulting H_2_O_2_ acted as the oxidation fuel for the HRP catalyzed oxidation of Amplex Red to the fluorescent Resorufin in the fused protocell containment (Figure 8C). The biocatalytic cascade operated only upon the fusion of the three liposomes and integration of the loads upon mixing the constituents in the fused protocell containments.

**Figure 9:**
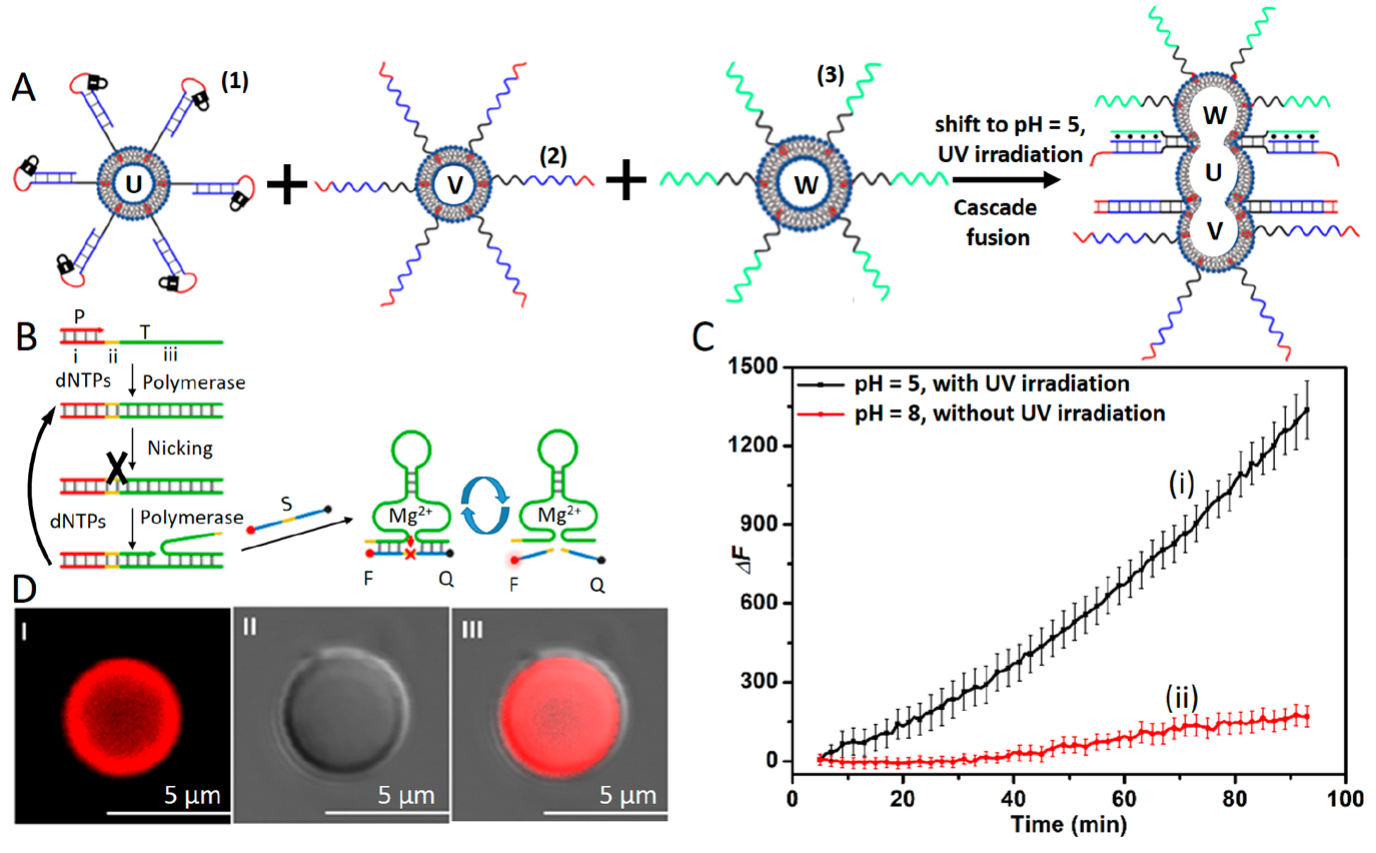
(A) Cooperative photoinduced and pH-triggered fusion of three liposomes U, V, and W using duplex and triplex fusion motifs, to yield a fused containment loaded with a polymerization/nicking DNA machinery. (B) Polymerization/nicking machinery for autonomous synthesis of the Mg^2+^-ion-dependent DNAzyme. The temporal emergence of the DNAzyme is followed by the DNA-catalyzed cleavage of the fluorophore/quencher modified substrate present in the fused liposome containment. (C) Curve i: fluorescence changes resulting upon the activation of the polymerization/nicking machinery in the fused three-liposome assembly and the emergence of the Mg^2+^-ion-dependent DNAyzme that catalyzes the cleavage of the fluorophore/quencher modified substrate. Curve ii, control system: treatment of the three-liposome mixture at pH = 8, without photoinduced activation of the system. (D) Confocal fluorescence microscopy images corresponding to (Panel I) the fluorescence of the fluorophore (TAMRA) generated in the fused three liposomes assembly upon operating the polymerization/nicking machinery and the subsequent DNAzyme-catalyzed cleavage of the fluorophore/quencher-modified substrate for a time-interval of 3 h, (Panel II) bright-field image of the fused liposomes, and (Panel III) overlay image of Panels I and II. Reprinted with permission under a Creative Commons CC BY License from ref (86). Copyright 2023, Wiley-VCH GmbH.

Figure 9A introduces the light/pH-triggered fusion of three liposomes and the integration of a dynamic DNA replication/nicking machinery in the fused protocell assembly.^86^ A mixture of three liposomes, U, V, and W, where U is modified at its boundary with the *o*-nitrobenzylphosphate ester photoresponsive caged nucleic acid hairpin structure (**1**) and loaded with DNA polymerase (Klenow), the nicking enzyme (Nt.BbvCI), and dNTPs. Liposomes V are functionalized with nucleic acid (**2**) and loaded with DNA template T that hybridized with promoter P (T/P), and liposomes W are functionalized with nucleic acid (**3**) and loaded with fluorophore (TAMRA) and quencher (BHQ2)-modified ribonucleobase functionalized substrate S. The liposomes were fused by light-stimulated deprotection of (**1**) at pH = 5.0. The fusion of the three liposomes proceeded by the concomitant deprotection of (**1**) and formation of the (**1′**)/(**2**) duplexes and (**3**)/(**1′**)/(**1″**) triplexes (cf. Figure 4D). The fusion process led to the mixing of the contents of the three liposomes and the activation of the DNA polymerization/nicking cascade in the fused protocell, leading to the autonomous synthesis of the Mg^2+^-ion-dependent DNAzyme in the protocell assembly (Figure 9B). The promoter/template (T/P) hybrids included in the template strand domain (i) that binds the promoter P, domain (ii) that encodes the instructive sequence that guides the nicking of the replicated domain by Nt.BbvCI, and domain (iii) that includes the complementary sequence to the Mg^2+^-ion-dependent DNAzyme. Accordingly, the fusion of the three liposomes stimulates the autonomous polymerase/nicking of the Mg^2+^-ion-dependent DNAzyme in the protocell. The resulting Mg^2+^-ion-dependent DNAzyme cleaves the fluorophore/quencher functionalized substrate S, and the fluorescence of the fragmented substrate provides an output signal for the temporal operation of the replication/nicking machinery (Figure 9C, curve i). A control experiment revealed that the nonfused separated liposomes did not lead to operation of the polymerization/nicking machinery (Figure 9C, curve ii). The operation of the polymerization/nicking machinery and the formation of the fluorescent fragmented product in the fused liposome assembly could be followed by confocal fluorescence microscopy imaging of a single protocell containment (Figure 9D).

**Figure 10:**
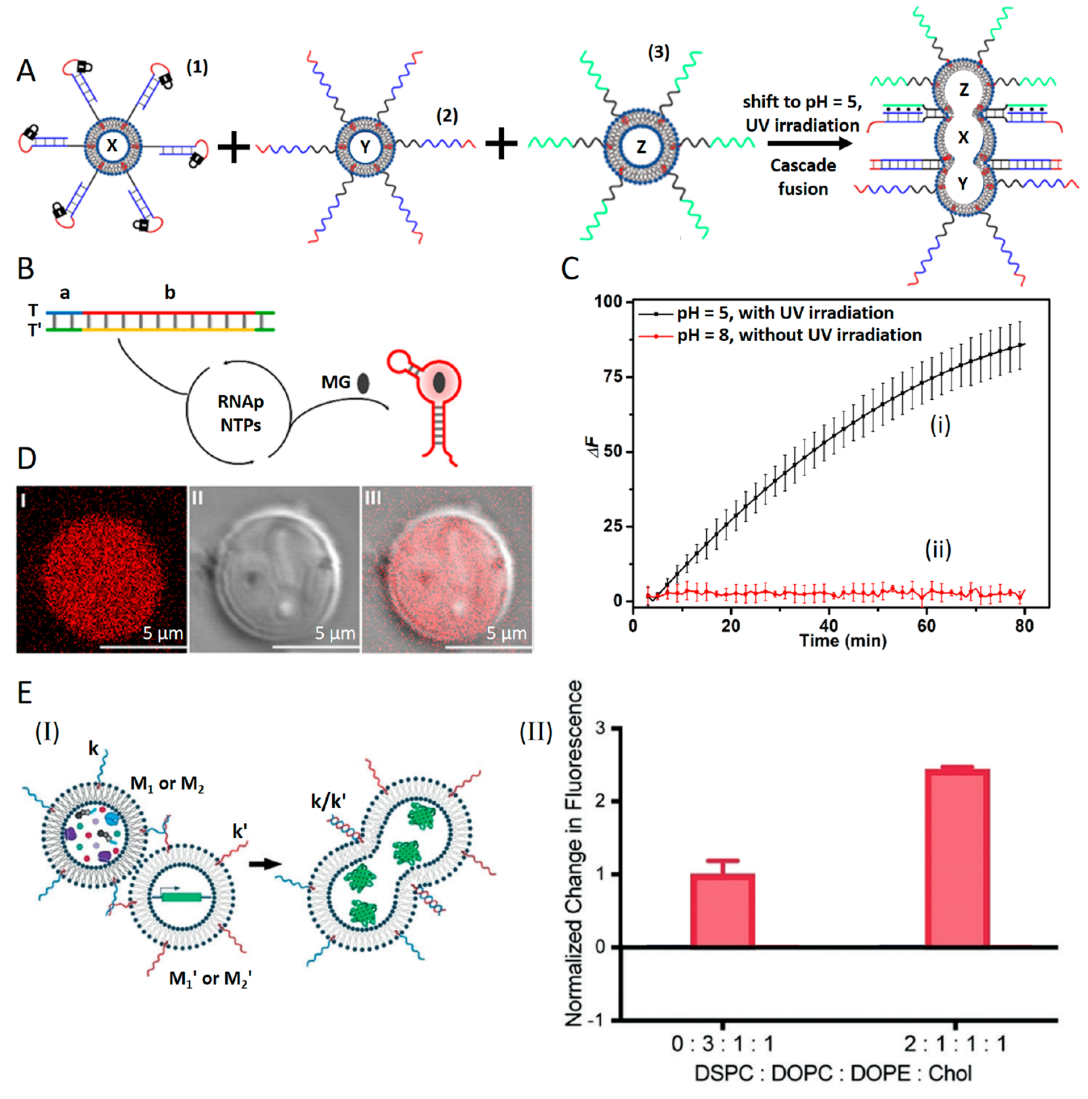
(A) Cooperative light- and pH-triggered fusion of three loaded liposomes X, Y, and Z using the duplex and triplex fusion motifs, where X (loaded with T7 RNA polymerase and NTPs), Y (loaded with T/T′ DNA templates), and Z (loaded with MG) yield a fused containment loaded with the transcription machinery that synthesizes the MG-RNA aptamer. (B) Transcription machinery guided circuit for the generation of the fluorescent MG/RNA aptamer complex in the fused containment. (C) Time-dependent fluorescence changes upon (i) the activation of the transcription machinery in the three-liposome fused containment and (ii) control fluorescence changes in the nonfused mixture of the three liposomes. (D) Confocal microscopy images corresponding to (Panel I) the fluorescent MG/RNA aptamer complex generated by the transcription machinery in the fused containment for a time interval of 3 h, (Panel II) bright-field image of the fused containment, and (Panel III) overlay image of Panels I and II. Reprinted with permission under a Creative Commons CC BY License from ref (86). Copyright 2023, Wiley-VCH GmbH. (E) Effect of bilayer lipid composition on the fusion of two liposomes loaded with the GFP plasmid and the cell-free expression kit, respectively, on the fusion efficiencies of the liposomes and on the resulting GFP expression yields in the fused containments. Reproduced with permission from ref (77). Copyright 2019, Wiley-VCH.

A further three-liposome-fusion process allowing the temporal operation of a transcription machinery expressing the Malachite Green (MG) RNA aptamer in a protocell containment is introduced in Figure 10A.^86^ A mixture consisted of three liposomes, X, Y, and Z, where liposome X was functionalized with the cholesterol-modified *o*-nitrobenzylphosphate ester photoresponsive hairpin structure (**1**) and loaded with T7 RNA polymerase (RNAp) and the NTPs. Liposome Y was functionalized with nucleic acid (**2**) and loaded with the transcription template T/T′, and liposome Z was modified with nucleic acid (**3**) and loaded with MG. The system was subjected to light (λ = 365 nm) at pH = 5.0. This led to the fusion of the three liposomes through the concomitant deprotection of the hairpin structure (**1**) and by formation of cooperative duplex (**1′**)/(**2**) and triplex (**3**)/(**1′**)/(**1″**) interconnecting bridges between the liposomes (cf. Figure 4D). The fusion of the three liposomes led to the mixing of the contents of the individual liposomes and the activation of the transcription machinery in the fused protocell containment (Figure 10B). The transcription template consisting of T/T′ included in the domain (b) the transcription sequence for the MG RNA aptamer, and thus the mixing of the contents of the three liposomes led to the RNAp-stimulated transcription of the MG RNA aptamer that binds the MG ligand to form the fluorescent MG/RNA aptamer complex. The temporal evolution of the MG/RNA aptamer complex in the protocell containment (Figure 10C, curve i) provided the readout output for the operation of the transcription machinery in the fused containment. A control experiment revealed that in the nonfused separated liposomes mixture, no transcription of the MG RNA aptamer occurred (Figure 10C, curve ii). The formation of the MG/RNA aptamer complex in the single fused protocell containment could be followed by confocal fluorescence microscopy (Figure 10D). Also, the lipid boundary composition of the interacting liposomes undergoing DNA-bridged fusion plays an important role in the fusion efficiency and the effectiveness of the reactivity within the fused containment. This is exemplified in Figure 10E with the fusion of loaded liposomes in which translation of proteins proceeds.^77^ In Figure 10E (Panel I) two liposomes M_1_ (or M_2_) and M_1_′ (or M_2_′) loaded with the green fluorescent protein (GFP) plasmid and the cell-free expression system (PURExpress), respectively, where the boundaries of M_1_, M_1_′ consisted of DOPC, DOPE, and Chol at a ratio of 3/1/1 and M_2_, M_2_′ composed of DSPC, DOPC, DOPE, and Chol at a ratio of 2/1/1/1 and functionalized at each set of liposomes with DNA tethers (k and k′), were fused to yield the load mixture leading to the translation of the GFP. While the fusion of M_1_/M_1_′ led to inefficient fusion and the translation of GFP, the fusion of M_2_/M_2_′ within phase-segregated domains led to a 2-fold enhanced fusion efficiency and translation of the GFP (Panel II).

## Fusion of Nucleic Acid Modified Liposomes with Cells

The delivery of auxiliary low-molecular-weight substrates or proteins into cells is an important step toward controlling cell functionality and cell viability. While the direct transport of molecular or macromolecular agents across the cell membranes is usually hindered, the development of means to overcome the cell boundary barriers may allow the transport of these agents, providing versatile methods to control cellular functionalities by guiding the transport of proteins or drugs that control cell functions. The fusion of nucleic acid modified liposomes with nucleic acid functionalized cells could provide a means for the guided delivery of loads integrated in the liposomes into the cells, leading to programmed and dictated cellular functions. The nucleic acid driven interconnection of the liposomes and cells, leading to fusion, might originate from internucleic acid bridges consisting of duplexes, triplexes, or quadruplexes or, alternatively, from duplexes stemming from aptamer strands linked to receptor ligands associated with the cells and complementary nucleic acid strands associated with the liposomes. The present section will address different methods to induce liposome–cell fusion for the guided delivery of auxiliary loads that control cell functionalities.

**Figure 11:**
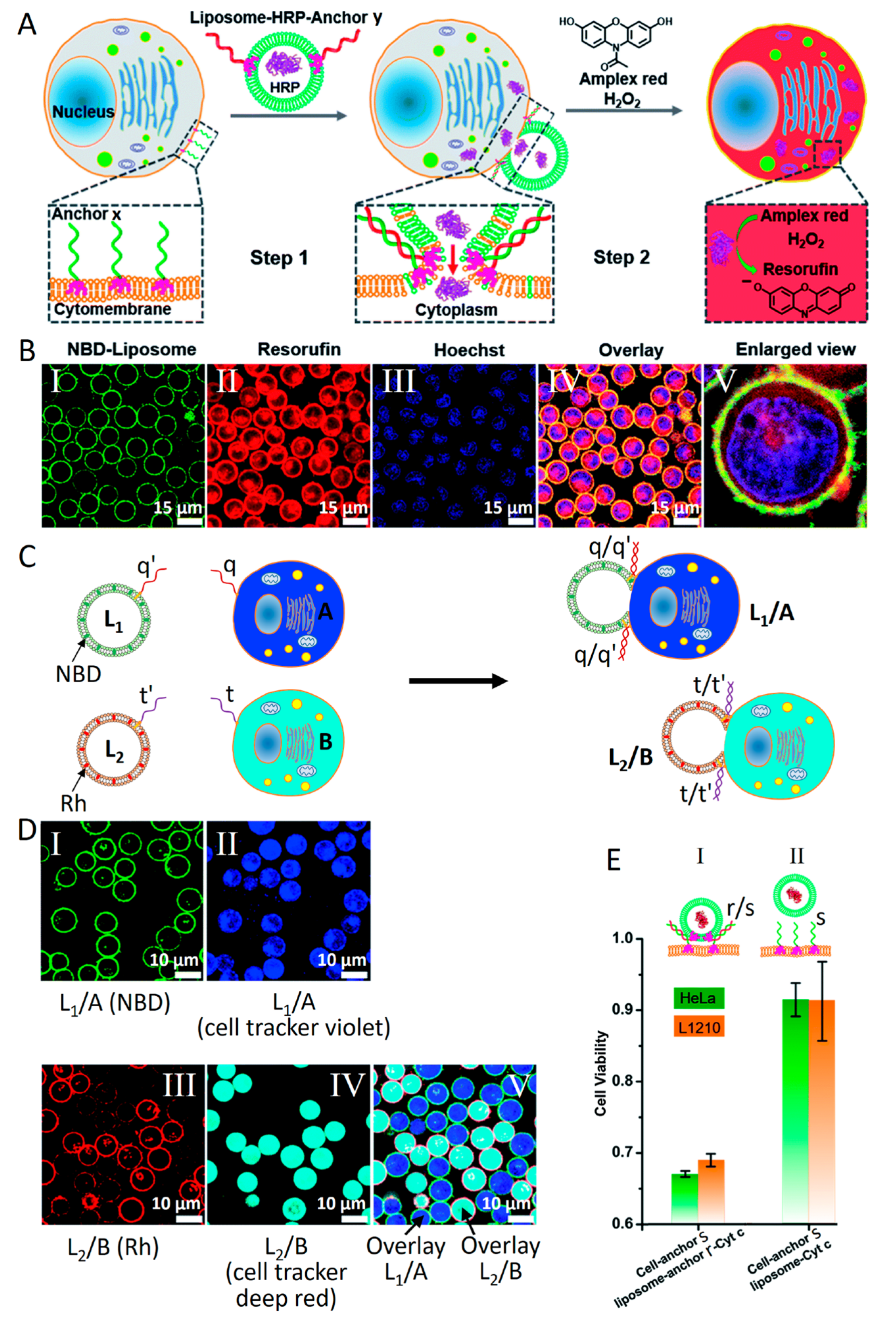
(A) Schematic fusion of a HRP-loaded nucleic acid (y) functionalized liposome with nucleic acid x modified L1210 cells (x/y complementary strands) and activation of the HRP-catalyzed oxidation of Amplex Red by H_2_O_2_ to the fluorescent Resorufin in the fused liposome–cell containment. The fusion process is followed by using confocal fluorescence microscopy imaging. (B) Confocal microscopy images corresponding to (Panel I) the fluorescence of NBD distributed in the liposome–cell fused hydrophobic boundary, (Panel II) the fluorescent image of Resorufin generated by the HRP driven oxidation of Amplex Red in the fused liposome-cell containment, (Panel III) staining the cell nucleus with the Hoechst dye and monitoring the fluorescence image through the Hoechst channel, (Panel IV) overlay image of Panels I–III, and (Panel V) enlarged overlay image of a single liposome–cell fused containment. (C) Selective nucleic acid guided fusion of two liposomes L_1_ (functionalized with nucleic acid q′ and labeled with NBD fluorophore in the boundary) and L_2_ (functionalized with nucleic acid t′ and Rhodamine fluorescent dye in the hydrophobic boundary), L1210 cells (A) modified with nucleic acid q (loaded with cell tracker violet), and L1210 cells (B) modified with nucleic acid t (loaded with cell tracker deep red). The sequence dictated complementarity of q/q′ and t/t′ leads to selective fusion of L_1_/A and L_2_/B. The selective fusion is followed by confocal fluorescence imaging. (D) Fluorescence confocal microscopy images of (Panel I) the NBD boundary labeled L_1_/A fused containment, (Panel II) the L_1_/A fused containment imaged through the cell tracker violet channel, (Panel III) the Rh boundary labeled L_2_/B fused containment, (Panel IV) the L_2_/B fused containment imaged through the cell tracker deep red channel, and (Panel V) overlay image of the mixture of L_1_/A and L_2_/B fused cells. (E) Probing Cyt c induced cell apoptosis by fusion of Cyt c loaded liposomes modified with nucleic acid r with HeLa/L1210 cells functionalized with nucleic acid s (nucleic acid r/s include complementary strands), where fusion leads to effective apoptosis (35% cell death), Panel I. A control system includes interaction of non nucleic acid modified liposomes with nucleic acid (s) functionalized with HeLa/L1210 cells. Lack of fusion leads to inefficient apoptosis (10% cell death), Panel II. Reproduced with permission from ref (99). Copyright 2018, Royal Society of Chemistry.

Figure 11A depicts the schematic integration of the enzyme HRP into a cell, e.g. L1210 cells, through the fusion of a HRP-loaded liposome with the cell.^99^ The cell membrane was functionalized with the cholesterol-modified nucleic acid x, and the HRP-loaded liposomes were modified with cholesterol-functionalized nucleic acid y, complementary to the strand x. The boundary of the liposomes was labeled with the hydrophobic fluorophore nitrobenzoxadiazole (NBD) for imaging purposes. Subjecting the cells to the liposomes resulted in their duplex x/y interlinkage, followed by the duplex x/y-mediated fusion of the liposomes with the cells. The fusion process led to the delivery of HRP into the cell cytoplasm and distribution of the NBD-fluorophore in the fused cell membrane boundary. Subjecting the fused cell–liposome assembly to Amplex Red and H_2_O_2_ resulted in the HRP-catalyzed oxidation of Amplex Red by H_2_O_2_ to form the fluorescent Resorufin dye in the fused cell cytoplasm. Furthermore, by Hoechst-dye staining of the nucleus of the cell (blue fluorescence), the fusion process could be followed by confocal fluorescence microscopy (Figure 11B). The fusion process resulted in the distribution of NBD in the fused cell boundary (Panel I). The Resorufin fluorescent product in the fused cell cytoplasm, upon the HRP-catalyzed oxidation of Amplex Red by H_2_O_2_, was followed by the red fluorescence channel of Resorufin (Panel II), and the Hoechst dye staining of the nucleus of the fused cell was followed by the blue fluorescence channel (Panel III). The overlay image of the fused cells is presented in Panel IV, and an enlarged overlay single cell image confirming the presence of all fluorescent labels associated with the fused assembly is shown in Panel V. The duplex nucleic acid-guided fusion between liposomes and cells enabled the selective fusion of liposomes with target cells. This is exemplified in Figure 11C, where a mixture of two liposomes L_1_ and L_2_ was subjected to two different L1210 cells, stained with cell tracker violet dye, functionalized with nucleic acid (q) (cell A) and with the cell tracker deep red functionalized with nucleic acid (t) (cell B), respectively. The liposomes L_1_ and L_2_ were modified with the cholesterol functionalized nucleic acid (q′) and (t′), and their boundaries were modified with the hydrophobic NBD and Rhodamine (Rh) dyes, respectively. The mixture of cells A and B in the presence of the liposomes L_1_ and L_2_ led to the selective duplexes q/q′ and t/t′ guided fusion of L_1_/A and L_2_/B, and the selective fusion processes were followed by confocal fluorescence imaging of the fused cells labeled with the respective fluorescent dyes (Figure 11D). The control over cell functionalities, as a result of liposome–cell fusion and delivery of the load from the liposome to the cell cytoplasm, is further demonstrated in Figure 11E. Cytochrome c (Cyt c) delivered from the cell mitochondria into the cell cytoplasm is known to trigger cell apoptosis.^100,101^ Accordingly, liposomes loaded with Cyt c and functionalized with cholesterol-modified strand r were fused with HeLa cancer cells or L1210 cells functionalized with the complementary nucleic acid s to yield the duplex r/s fused liposome–cell assemblies, and the Cyt c induced apoptosis of the cells was probed (Figure 11E). While the Cyt c-treated fused cells demonstrated effective apoptosis (ca. 35% cell death, Panel I), the cells fused with nonanchor Cyt c containing loads demonstrated substantially lower apoptosis (ca. 10% cell death, Panel II).

**Figure 12:**
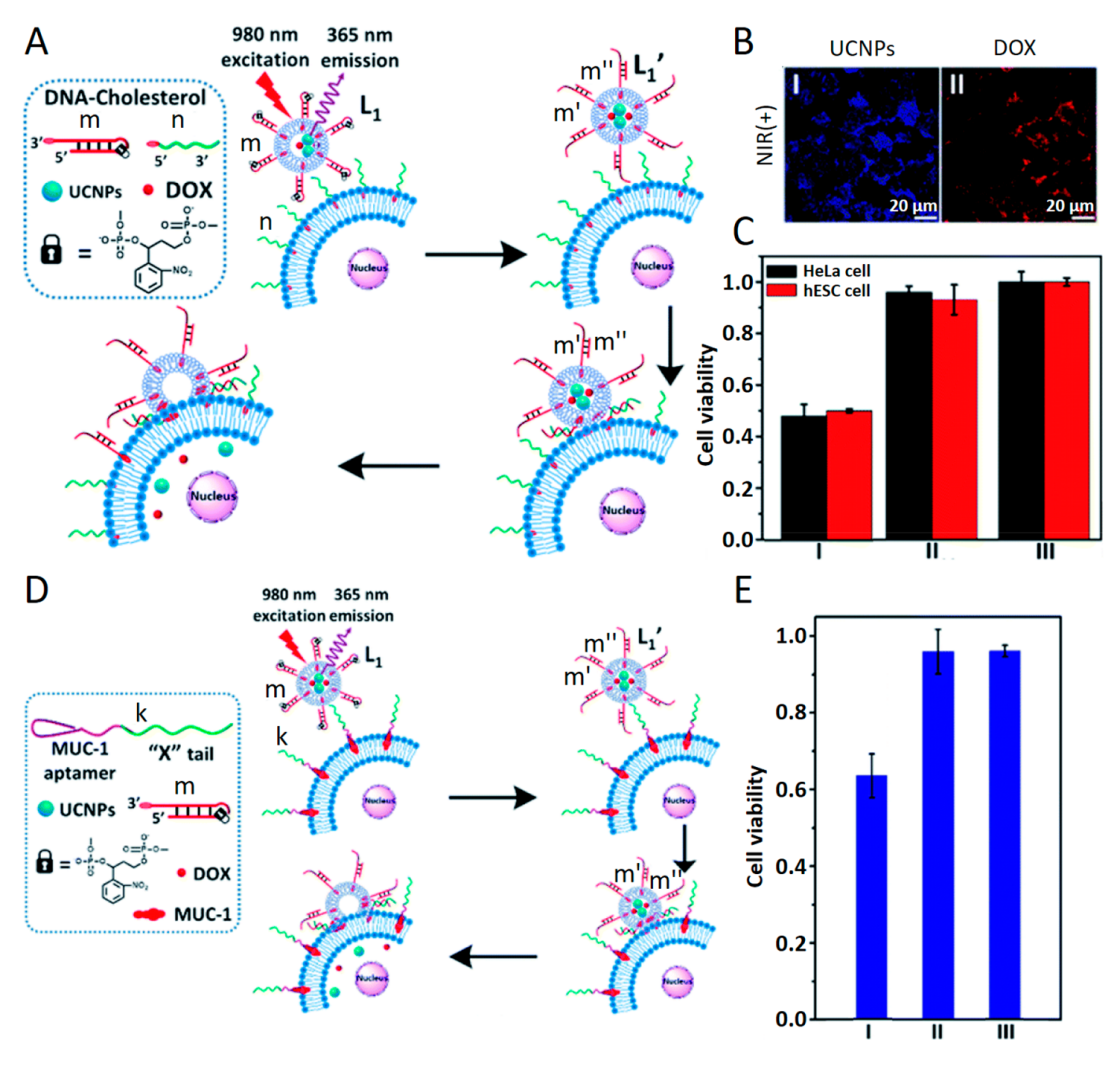
(A) Photoinduced-triggered fusion of *o*-nitrobenzylphosphate ester hairpin functionalized liposomes loaded with UCNPs and DOX with nucleic acids functionalized HeLa cancer cells. Photoexcitation of the UCNPs (λ = 980 nm) results in UCNPs-stimulated photodeprotection of the hairpins and fusion of the liposomes with cells using the duplex fusion motif, resulting in the delivery of the DOX drug into the cells. (B) Confocal fluorescence images corresponding to (Panel I) the UCNP modified fused liposomes/cells (blue channel) and (Panel II) the fluorescence of the DOX-loaded fused liposome–cells (red channel). (C) Cytotoxicity of the DOX/UCNP-loaded liposomes toward HeLa/hESC cells upon (I) NIR light-induced fusion of the liposomes with the cells and delivery of the drug into the cells, (II) cells treated without photochemical-stimulated fusion, (III) Nontreated cells. The cell viability was evaluated after a time interval of 2 days of treatment. (D) Targeted photochemical fusion of MUC-1 aptamer modified HeLa cells and *o*-nitrobenzylphosphate ester hairpin functionalized liposomes loaded with DOX and UCNPs. (E) Cytotoxicity of the liposomes described in (D) toward the HeLa/hESC cells upon (I) the photoinduced fusion of the liposomes with the HeLa cells, (II) photochemical fusion of liposomes lacking the DOX drug with the HeLa cells, (III) hESC cells lacking the MUC-1 receptors treated with the photoresponsive liposomes shown in (D) under light. Cytotoxicity was evaluated after 2 days of treatment. Reprinted with permission under a Creative Commons CC BY 3.0 License from ref (44). Copyright 2020, Royal Society of Chemistry.

The application of light as a trigger to induce liposome–cell fusion is particularly attractive, as it allows the spatiotemporal activation of the fusion process and the dictated release of loads (e.g., drugs) into the cells.^44^ However, the use of the *o*-nitrobenzylphosphate ester photoactive group to uncage the nucleic acid functionalities toward the fusion process (cf. Figure 4) requires UV light (λ = 365 nm), which is harmful toward cellular environments. Thus, to allow the application of *o*-nitrobenzylphosphate ester modified nucleic acid photoactive constituents to stimulate the liposome–cell fusion process, switching the light source to the visible or NIR region to uncage the photoactive groups is desirable. Different methods to switch the photodeprotection processes of these materials were suggested, including the modification of the *o*-nitrobenzyl moieties with red-shifting electron donor substituents,^102−104^ the application of two-photon laser excitation,^105−107^ or the use of UCNPs excited in the NIR region.^108−110^ Indeed, UCNPs were employed to deprotect *o*-nitrobenzylphosphate ester nucleic acid functionalities associated with liposome–cell fusion by applying NIR light sources.^44^ In these systems, the excitation of UCNPs by a 980 nm light source yielded localized fluorescence at 365 nm that acts as a light source for the deprotection of the *o*-nitrobenzylphosphate ester caged nucleic acid units. This is exemplified in Figure 12A^44^ with the UCNP stimulated fusion of doxorubicin (DOX)-loaded liposomes with HeLa cancer cells, resulting in the spatiotemporal controlled delivery of DOX into cancer cells. Liposomes L_1_ functionalized with cholesterol-modified *o*-nitrobenzylphosphate ester caged hairpin nucleic acid m, loaded with DOX and the UCNPs, were fused with HeLa cells modified with cholesterol-functionalized nucleic acid n. The liposome–cell mixture was subjected to NIR irradiation (λ = 980 nm), resulting in the photodeprotection of the hairpin constituent associated with the liposome L_1_ to yield the m′/m″ duplex modified liposome boundaries. The displacement of the duplex constituents m′/m″ by the strand n, associated with the HeLa cell, resulted in the m′/n bridged interconnection of the liposomes and the cells and the subsequent fusion of the liposomes with the cells. This resulted in the delivery of the liposome-loaded DOX and UCNPs into the cells. The delivery of the loads was imaged by confocal fluorescence microscopy following the fluorescence of the UCNPs or DOX by the respective imaging channels (Figure 12B). The delivery of DOX into the HeLa cells (or hESC epithelial cells) demonstrated the nonspecific cytotoxicity of the delivered chemotherapeutic agents toward the two kinds of cells (Figure 12C). After a time interval of 2 days, ca. 60% of cell death of the two types of cells was observed, while no cytotoxic effect of the DOX-vacant, or nonirradiated liposomes, preventing liposome fusion to the cells, was detected. To overcome the nonselective cytotoxic effect demonstrated by the DOX-loaded liposomes, the fusion system was modified by targeting the liposomes to the cancer cells using specific aptamer–ligand interactions guiding the selective fusion of the DOX-loaded liposomes with the cancer cells (Figure 12D). The HeLa cancer cells, functionalized at their cell membranes with the MUC-1 receptor units, were modified with the MUC-1 aptamer strand extended with domain X in strand k. The UCNPs and DOX-loaded *o*-nitrobenzylphosphate ester m modified liposomes were subjected to the k-modified HeLa cells and irradiated at 980 nm to yield the UCNP-stimulated photodeprotection of m-functionalized liposomes to the m′/m″-modified state. As the sequence of domain “X” associated with the k strand was designed to be complementary to m′, strand displacement of the m′/m″ duplex by k led to bridging the liposomes with the HeLa cells, leading to their fusion and the delivery of the DOX load into the HeLa cancer cells. Note, however, that since the normal hESC lacked the MUC-1 receptor in their boundaries, they could not be functionalized with the aptamer specific strand k, and thus, the light-stimulated fusion with the m-functionalized DOX/UCNPs loaded liposomes was prohibited. Indeed, selective cytotoxicity was observed upon the light-stimulated fusion of the k-modified HeLa cells with the m-functionalized DOX/UCNPs loaded liposomes (Figure 12E). While the light-induced fusion of the DOX/UCNPs-loaded m-functionalized liposomes with the k-functionalized cells led to 40% cell death after 2 days, no cytotoxic effect was observed upon light-stimulated treatment of the hESC cells with the drug-loaded liposomes.

## Conclusions and Perspectives

The significance of the recognition features and triggered reconfiguration functions of nucleic acids for guiding the fusion of liposome and membrane-like interfaces were introduced. The nucleic acid driven contact between the interfaces provided tools to initiate fusion and the exchange of loads between the cell-like containments. The biophysical constraints involved with the nucleic acid guided fusion of liposomes were addressed. The programmed multifusion of liposomes and the exchange of payloads and functional constituents upon fusion enabled the operation of gated and cascaded biocatalytic transformations. The triggered light-induced fusion of liposomes functionalized with *o*-nitrobenzylphosphate ester photoresponsive groups and particularly the application of upconversion particles to induce the fusion by NIR irradiation were introduced. These concepts allow selective and spatiotemporal fusion and drug delivery into cells. Besides highlighting the advances in developing artificial cells by the fusion of nucleic acid–based liposomes and membrane interfaces, this review emphasizes the future perspectives of the field: (i) While different structural motifs of DNA, such as duplex or triplex bridging of the membrane interfaces, provided means to stimulate the fusion, other reconfiguration paths of nucleic acids such as metal-ion-bridged nucleic acids,^38,39^ aptamer–ligand complexes,^111,112^ or duplex nucleic acid bridges stabilized by photoisomerizable intercalator (e.g. *trans*-azobenzene) can be envisaged.^41−43^ (ii) While the feasibility of integrating biocatalytic machineries into fused liposomes through exchange of loads was demonstrated and cell functions such as replication or polymerization were emulated, the integration of functional reaction scaffolds and particularly dynamic frameworks triggered by auxiliary stimuli is particularly challenging. For example, the incorporation of triggered biocatalytic constitutional dynamic networks^113^ or fueled dissipative transient reaction moduli^114^ are interesting directions to follow. (iii) The selective and targeted fusion of liposomes with living cells and the introduction of auxiliary loads into the cells was demonstrated. However, the delivery of payloads of enhanced complexities, particularly molecular machines that intervene with cellular pathways, is anticipated to establish revolutionary therapies. (iv) The discussion focused on the fusion processes and the advantages of payload mixing and the functional output of the mixed containments. The most challenging issue is, however, the development of means to separate the fused containments into subcontainments that comprise the functions of the fused compartments. This could provide model systems for cell division and proliferation. Toward this goal, we note that the fusion process involved reconfigurable nucleic acid bridges. These sites might act as “hot spots” for the separation of the fused containments. Thus, future exciting biophysical research into membrane fusion and emergent cell-like functions is envisaged.

## Vocabulary

**Nucleic acid induced liposome fusion**: Liposomes are synthetic globular membrane-like cell mimicking containments self-assembled through the aggregation of amphiphilic constituents. The liposomes can be functionalized with programmed loads. Integration of amphiphilic nucleic acids into the liposome membranes yields functional stimuli-responsive liposomes allowing nucleic acid guided fusion of liposomes and exchange of loads to occur.
**Biophysical insight into nucleic acid-guided fusion of liposomes**: The structural engineering of nucleic acid tethers guiding the fusion of the liposomes, particularly nucleic acid tendril structures, plays an important role in the liposome fusion efficiencies. The bilayer environment of the nucleic acid bridging units affects the fusion yields and the quality of the fused containments.
**Photodeprotected caged nucleic acid structures**: *o*-Nitrobenzylphosphate ester modified caged amphiphilic constituents in liposome membranes are photochemically uncaged by UV light (λ_ex_ = 365 nm) or the near-IR application of upconversion nanoparticles. The uncaged nucleic acid constituents provide functional units to induce liposome fusion.
**Physical and spectroscopic tools to probe liposome fusion**: Diverse physical and spectroscopic methods to probe liposome fusion are employed, including dynamic light scattering, scanning electron microscopy, fluorescence confocal microscopy, and dynamic photochemical probing of quenched fluorescent dyes upon fusion.
**Biocatalytic transformations in protocells**: Protocells are structural containments emulating functions of native cells. By fusion of loaded liposomes, integrated protocell assemblies stimulating biocatalytic cascades or DNA machineries are demonstrated.
